# Simulating the Effect of Mixed Subsidy Policies on Urban Low-Value Recyclable Waste in China: A System Dynamics Approach

**DOI:** 10.3390/ijerph182010636

**Published:** 2021-10-11

**Authors:** Dongshi Sun, Danlan Xie, Peng Jiang, Jingci Xie, Yang Xu, Yining Ren

**Affiliations:** 1 School of Information and Business Management, Dalian Neusoft University of Information, Dalian 116023, China; sundongshi@neusoft.edu.cn (D.S.); xuyang@neusoft.edu.cn (Y.X.); 2 Hangzhou College of Commerce, Zhejiang Gongshang University, Hangzhou 311599, China; xdl@zjhzcc.edu.cn; 3 School of Business, Shandong University, Weihai 264209, China; renyining@gmail.com; 4 School of Management, Shandong University, Jinan 250100, China; xjc@sdu.edu.cn

**Keywords:** subsidy policy, low-value recyclable waste, waste disposal, multiple subjects, system dynamics

## Abstract

Low-value recyclable waste accounts for a large portion of urban waste output in many modern cities. The improper management and disposal of LVRW result in environmental pollution and a waste of resources. Given the characteristics of a high recovery cost and low recovery income of low-value recyclables, it is difficult to obtain a satisfactory waste disposal effect by completely relying on the market mechanism. It is thus necessary for the government to implement effective subsidies for multiple subjects in the urban waste recycling system (UWRS). This study examines the independent roles of four subsidy policies—subsidy to the third-party waste disposal institutions, subsidy to a state-owned waste disposal institution, R&D subsidy for green technology, and subsidy for government publicity—and develops a system dynamics model to verify the performance of the UWRS under different combinations of subsidy-based policies under multiple scenarios. Data on urban waste disposal for Guangzhou from 2019 and 2020 were used to validate and simulate the model. A sensitivity analysis of the main exogenous variables was carried out, and the conclusions are as follows: (1) On the premise of a fixed subsidy capital pool, a mixed subsidy policy produced the best impact on the UWRS. (2) The total subsidy needed to reach a certain threshold; otherwise, the mixed subsidy policy did not improve the UWRS. The total subsidy produced diminishing returns once it had exceeded the threshold. (3) Appropriately reducing subsidies for the third-party waste disposal institutions within a reasonable range does not affect the performance of the UWRS. (4) The effect of government publicity has short-term advantages, while the long-term potential of green technology is greater. Multi-agent coordination and the guidance of the market mechanism are important priorities in the design of subsidy-based policies. In addition, the trade-off between subjects needs attention, and a plan for mixed subsidy policies needs to be designed and implemented according to the response periods of different policies. The research here provides theoretical support for the government for designing subsidy-based policies.

## 1. Introduction

With accelerating urbanization and economic development across the world in recent decades, the urban waste output has been increasing rapidly. However, many cities still use the traditional modes of waste disposal, of burning and burying, which does not make full use of the resources within the urban waste and causes severe environmental pollution. In tackling the problem of urban waste disposal, countries have adopted different models according to their national conditions. For example, Germany enforces the Extended Producer Responsibility (EPR) [1] and has established Duales System Deutschland (DSD) that organizes the recycling of packaging waste [2]. It trusts the relevant companies to recycle and classify their packaging waste and send it to the corresponding resource reuse factory for recycling. Packaging waste that can be directly recycled is sent back to the manufacturer [3]. In Brazil, the CEMPRE model is used [4], whereby waste collectors in the city are organized into cooperatives and trained in waste classification. After classification, the waste is packaged, compressed, and sold to enterprises registered with CEMPRE [5,6]. These models have achieved good results in their respective countries, but they also have clear limitations, that is, the recycling value of urban waste must be high enough to draw multiple subjects to participate in waste disposal.

In many countries, urban residents usually sell the high-value part of waste [7] that they produce owing to poor living standards, the composition of the waste, and its mode of discharge. Once the waste has been discharged, scavengers rake more carefully through it such that almost no medium- or high-value recyclables remain in it [8], but it contains a large amount of low-value recyclable wastes (LVRWs) [9]. LVRW refers to solid waste, such as waste glass, wood, flexible packaging, and plastics [10]. They have recycling value, but the cost of recovering them is higher than this value [11]. Therefore, LVRWs are difficult to recycle in the market mechanism. They cannot be accurately traced to the producer either [12], because of which EPR cannot be implemented. 

In China, the average ratio of LVRW to urban waste is 38% [8]. In the past 20 years, state-owned waste disposal institutions (SOs) were mainly responsible for waste disposal in China. The simplification of the mode of waste disposal resulted in increasingly serious environmental problems [13]. In recent years, the government has encouraged the interventions by the third-party waste disposal institutions (3Ps) through subsidies [14], but this has not yielded a significant improvement in waste disposal while imposing a significant financial burden on the government [15]. This is because the current subsidy to 3Ps in China is settled according to the amount of disposed waste [10]. This form of subsidy cannot promote 3Ps to actively improve the waste recovery rate, nor reduce the waste disposal costs of 3Ps [16]. On the contrary, it will lead to dishonest behaviors such as falsely reporting the waste disposal capacity.

In cities with a high ratio of LVRW, the subsidy policy should not only provide support for the basic operation of waste disposal institutions but should also help improve the recycling rate of LVRWs to gradually replace public welfare operation with the market mechanism, establish an urban waste recycling system (UWRS), and build mechanism of synergy between multiple subjects in the UWRS. 

Some classic methods were used in the synergy mechanism field. When the number of subjects in a system is not more than three, the evolutionary game theory was widely used to analyze the equilibrium strategies between multiple subjects [17,18,19], but there are at least five subjects in the UWRS, including the government, the SO, 3Ps, city dwellers and research institutions. The relationships between subjects are too complicated for evolutionary game methods. Operations research models are other popular methods in this field [20,21], but in the UWRS, the objectives of each subject are inconsistent, the total objective function in the UWRS is not easy to set and constraints and decision variables are too many, making the model difficult to solve. Although qualitative analysis methods such as case analysis and grounded theory can also design synergy mechanisms, they are generally used for macro-analysis and cannot solve the practical problems in this paper. System dynamics (SD) is an effective tool for looking for the root cause(s) of a given problem based on the internal structure of the system according to the feedback characteristics of its components [22]. In this article, the cause of the problem of urban waste disposal in China can be identified and the logical relationships and interactions among the subjects in the UWRS and the impact of variables on its performance can be easily explained by using the SD model. In addition, SD can simulate the performance of the system when multiple variables in it change at the same time, and dynamically optimize the effects of mixed subsidy policies [23]. Therefore, the approach employed in this study is the SD method. The first aim of this paper is to construct a UWRS model with multiple subjects and study the effects of mixed subsidy policies on the UWRS under multiple scenarios. The second aim is to adjust the participation ratio of SOs and 3Ps in the UWRS to create a mechanism of positive competition between waste disposal institutions by redesigning the subsidy program. The third aim of this paper is to formulate a plan for the implementation of the mixed subsidy policies by identifying the response times of different policies and the intensities of their impacts on waste management.

The main contributions of this study are threefold: (1) This paper focuses on the recycling of LVRWs and enriches the theory of urban waste management. It reveals the relationship between the utilization rate of LVRW and the effect of urban waste management, providing a new perspective for solving the problem of urban waste management in China and a theoretical basis for government to issue law and regulations on LVRW. (2) By simulating an SD model, this paper defines the role of the individual subsidy policy on the UWRS and verifies the comprehensive effects of mixed policies. The conclusions provide support for decisions on the implementation of precise incentives. (3) This paper limits the government’s subsidy capital pool (SCP). The implementation of mixed subsidies is premised on not adding additional government financial burdens, which should pay attention to the allocation of subsidies among the subjects. Its policy implications are not only scientific in the context of theory but also feasible in practice.

The remainder of this article is organized as follows: Section 2 presents a literature review from the three perspectives of the mode of waste disposal, and the incentive mechanisms and subsidy policies for it. Section 3 describes the proposed SD model, and Section 4 reports the results of simulations of it for different subsidy policies as well as its impact on the UWRS of the main exogenous variables. Section 5 discusses the policy implications of this study and adaptations of the proposed model, and Section 6 provides the conclusions of this study.

## 2. Literature Review

### 2.1. Researches of LVRW 

LVRW is mentioned in the many literatures of different countries such as China [23], Czech Republic [24], India [25] and Canada [26]. It is also explained in the Measures for the Administration of the Purchase of LVRW Services in Guangzhou [10], which clarifies its importance and difficulties in the recycling of URWS. LVRW refers to solid waste, such as waste glass, wood, flexible packaging and plastics, which has low recycling value. It is difficult for enterprises to achieve income and expenditure balance by recycling LVRW [10]. However, enterprises are profit-driven and market regulation alone cannot make LVRW enter the recycling channel well. The current studies on LVRW are mainly focused on improving recycling rates and optimizing the LVRW recycling system. Xie [27] proposed that in the process of promoting the recycling of LVRW, technologies such as the Internet of Things and big data can be used to establish a big data cloud platform to promote waste sorting. Struk [24] analyzed that curbside collection improves LVRW separation rates by 40% compared to drop-off sites. These studies showed that the waste classification and waste collection mode were very important to improve the recovery rate of LVRW. However, these studies did not consider the residents’ awareness of recycling. If residents do not have the awareness of recycling, the effectiveness of waste collection mode would be greatly reduced. Therefore, not only waste collection mode but also residents’ recycling awareness should be considered when improving the LVRW recycling system. Ioannis [28] claimed that to fulfil the LVRW recycling targets (55%), notable improvements are necessary at the plants, product design, collection system and market level. This study considered the function of the waste collection system but ignored the importance of waste processing system. Effective disposal process can increase the recycling rate of LVRW [10]. Therefore, waste disposal process should be considered when improving the LVRW recycling system. Mahavadi et al. [25] proposed four policy interventions (charging disposal fees, provision of recycling subsidies, provision of curbside recycling facilities and a plastics ban), and explored the effects of these policy interventions. Du et al. [29] analyzed that different types of low-value recyclables should be implemented with gradient subsidy, and precise subsidies should be provided to enterprises to promote the industrialization of the industry. These studies explored the effectiveness of subsidies to improve the LVRW recycling system. However, these studies only considered the recycling companies but ignored the other participations in the LVRW recycling system. It cannot make the LVRW system achieve the best performance.

Our literature review reveals that in order for a LVRW recycling system to achieve the best performance, a suitable waste management model should be selected and multiple subjects in the LVRW recycling system should be considered.

### 2.2. Waste Management Model

Many countries now attend to urban waste management and consider waste a resource. New concepts in waste management, such as the “waste-free city,” are becoming popular. Nelles et al. [30] found that the EPR had been applied in Germany to allocate waste disposal responsibilities to manufacturers and distributors. Both the advanced disposal technology and the EPR can improve recycling capability. Pincelli et al. [31] studied the CEMPRE model used in Brazil and found that incorporating informal waste managers and producers from society into the recycling system can increase the recycling rate. Chifari et al. [32] claimed that the cost of the UWRS can be reduced in the following ways in Japan: the recyclable part of the waste is recycled separately, private companies offer their services through tendering, and adjacent municipal departments cooperate with one another. Reijonen et al. [33] investigated the problem of recycling plastic bottles in Finland and designed a deposit-based return system. They claimed that source separation was the key to improving the packaging of recycled plastic. Ceschi et al. [34] showed that promoting normative policies can increase recycling activities in Taiwan. Sukholthaman et al. [35] proposed that for the sustainable development of urban waste management in Thailand, close cooperation among stakeholders was important. They also claimed that the public–private–community partnership has high implementation value. Phonchi-Tshekiso et al. [36] tested the results of public–private cooperation to improve the capacity of solid waste management services in Botswana and verified the advantages of privatized waste management. Du [37] established a collaborative model for classifying waste led by the government, and involving participation by enterprises and communities, and showed that it can increase private resource recovery enterprises in the UWRS. 

The above research has shown that it is important to design a targeted waste management model according to the characteristics of the urban waste. However, few studies have considered LVRWs. Moreover, the participation of multiple parties, including the government, producers, 3Ps, non-governmental organizations, and community residents, can help reduce the cost of waste disposal, increase the recycling rate, and improve the performance of the UWRS. A conflict of interests among multiple subjects can easily lead to a failure of coordination. It is this important to design an incentive mechanism to balance the interests of subjects. According to the characteristics of the LVRW, the mechanism of incentives should be led by the government, which has the power to formulate policies and impose taxes. It can thus combine economic and administrative means in this context.

### 2.3. Incentive Mechanism for Waste Management

There are implementations of incentive mechanism for waste management. Table 1 summarizes and compares recent studies on the subjects of incentive and the relevant measures with previous studies.

Our literature review reveals that among these incentive mechanisms the subsidy mechanism is more suitable for LVRW before the LVRW recycling system operating well. The table above compares the incentive mechanisms for recycling LVRW with other recyclable waste. It is found that the main incentive subjects include residents, manufacturing companies, recycling companies and 3Ps. These subsidy measures can effectively improve the waste recycling rate. There are few incentive measures for manufacturing companies in recycling LVRW because LVRW cannot be traced back to its manufacturers accurately. Therefore, subsidies to manufacturing companies are not considered in the research of this paper. Scientific research institutions are rarely subsidized in current studies. However, technology is productivity [47] and technical improvement can improve the governance effect of LVRW [48]. The subsidy to scientific research institutions should be considered in the research of this paper.

The studies above focus on the application of subsidy mechanisms for LVRW recycling. However, each study only considered a single subsidy. Research on the mixed implementation of multiple subsidy mechanisms is inadequate. The UWRS contains a large amount of LVRW. In the URWS, the SO and 3P are responsible for waste disposal, scientific research institutions are responsible for upgrading technologies to improve the rate of waste recovery, and residents are responsible for classifying waste to reduce the cost of waste disposal. It is difficult to coordinate the operation of all subjects by relying on a single subsidy mechanism. Therefore, the mixed implementation of multiple subsidy policies for different subjects of subsidy need to be researched. The implementation of mixed subsidies should consider the urban environmental conditions and implementation conditions at different stages. Subsidies have different emphasis at different stages, and the current research are mainly static subsidies. Therefore, it is necessary to conduct a dynamic study on the subsidies of each subject.

## 3. Methodology

### 3.1. Introduction to SD

SD was developed in 1956 by J. W. Forrester at MIT. It is a simulation method that was originally used to analyze enterprise problems, such as production and inventory management. At present, SD is widely used in many fields, such as corporate management [49], urban planning [50], environmental [51] and agricultural development [52], construction engineering [53], and logistics supply chains [54,55].

For waste management, Dhanshyam et al. [25] established SD models to study plastic waste mitigation in India, and analyzed the effects of major policy interventions on plastic waste stock; Liu assessed effects of government reward and punishment on the recycling efficiency of waste batteries in China [56]; Saroj explored the obstacles in reverse logistics of packaging materials in the view of extended producer responsibility [57]; Pablo analyzed policies’ effects on the legal dismantling and recycling of electronic waste in Brazil [58]. Nonetheless, a systematic analysis of the effects of multiple subsidy policies on the UWRS with multiple subjects has not yet been conducted, nor has the dynamic recovery rate of the LVRW under the influence of multiple subsidy policies been investigated.

In light of the above, this paper establishes an SD model to simulate the performance of the UWRS in case of a large amount of LVRW under the influence of mixed subsidy policies. The modeling process of the SD follows the classic steps [59]. Step I: Investigate problems and policy implementations for the UWRS in China. Step II: Define the boundary and sub-modules of the system and establish a causal loop diagram (CLD). Step III: Establish the mathematical relationship between the variables, define the parametric equation based on the CLD, and establish the stock-flow model. Step IV: Conduct an authenticity test to validate the effectiveness of the model. Step V: Design different mixed incentive mechanisms, simulate them, and analyze the results to provide support for the formulation and implementation of policies.

### 3.2. Model Hypotheses

Based on the objectives of this study, the following hypotheses are proposed:

(1) The urban waste output per unit time is a fixed value. Waste output is affected by temperature, weather, urban economy, residents’ awareness of environmental protection, and other factors in the short term, and by living habits and the GDP in the long term. It is a value that conforms to the average distribution in the short term and grows linearly in the long term. It is set as a constant to eliminate interference by fluctuations in the waste output to compare the effect of differences of urban waste disposal under different policy combinations.

(2) The total subsidy capital pool of the government for urban waste disposal is fixed, but the specific ratio of each subsidy policy varies. Most studies on government subsidies do not restrict the total amount of subsidies. A subsidy policy may thus be effective but incurs a significant financial burden on the local government when implemented. The SCP is set as fixed to test the impact of policies in different proportions. At the same time, when the total amount of SCP remains unchanged, the final optimization scheme is highly applicable. 

(3) Because a large investment is needed to improve the rate of waste recycling, it is assumed that the 3P does not take the initiative in the R&D of green technology (GT), and its recycling rate is fixed. The government chooses to cooperate with 3Ps that meet specific standards related to the recycling rate. It supports the R&D of GT in the SO through subsidy policies. With the development of GT, the advanced waste disposal capacity of the SO improves, and the proportions of advanced and conventional waste disposal capacities continue to change, because of which the recycling rate of the SO changes over time. It is useful to verify the impact of subsidies for GT on system performance through this hypothesis.

### 3.3. Main Modules

**Figure 1:**
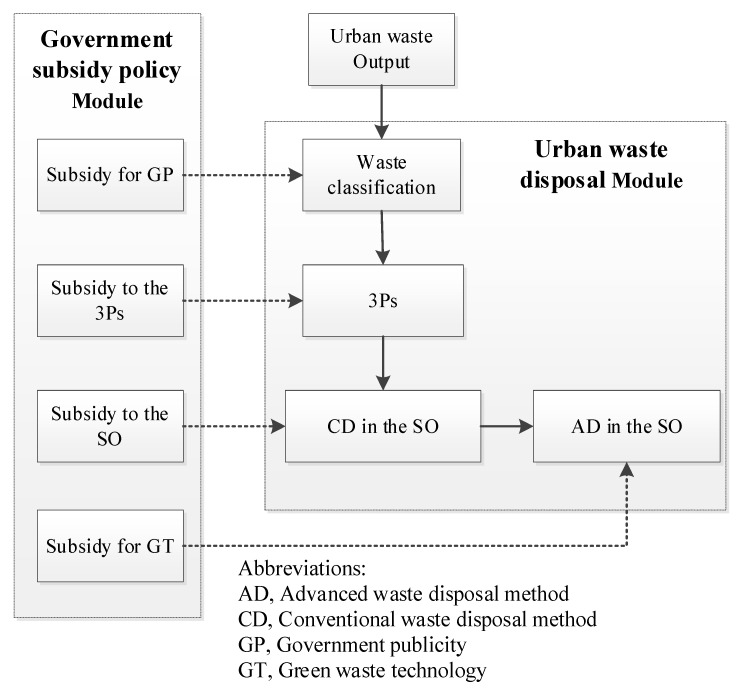
The model framework.

This paper applies the SD method to establish a UWRS model in the case of mixed subsidy policies. The model includes an urban waste disposal module and a government subsidy policy module. The urban waste disposal module consists of waste disposal modules of the 3P and the SO. The government subsidy policy module contains four policies that affect different processes of the urban waste disposal modules. The model framework is shown in Figure 1. This paper uses Guangzhou, a representative city in China, as an example to illustrate the modules. Guangzhou’s average waste output ranks among the top three cities in China. Burning and burying are the main modes of waste disposal, and the recycling rate of waste is low. The city is thus facing a serious problem of waste siege. The Guangzhou government has set-up subsidies for 3Ps and encouraged them to assist in waste disposal. However, the effect has been poor, and the financial pressure on the government is significant. It is eager to find ways to improve urban waste disposal.

#### 3.3.1. 3P Waste Disposal Module 

The high proportion of LVRW in China’s urban waste leads to the failure of the DSD model (from Germany) and CEMPRE (from Brazil) when they are applied to it. At present, 3Ps’s profit in waste disposal is low or even negative, so the intention of the 3P to engage in waste disposal completely depends on the amount of government subsidies. On the premise of limited government subsidies, 3Ps has the limited intention and can only be a supplement to the SO in terms of urban environmental governance at this stage. Therefore, reducing the operating cost and improving the income of waste disposal of 3Ps are the main ways to improve the intention of the 3P to engage in waste disposal [60].

The most important variable in this module is the intention of the 3P to engage in waste disposal. The relationship between this intention and the unit profit is fitted to an exponential function. To describe the relationship more accurately, questionnaires were sent to eight 3Ps that met the standard qualifications in Guangzhou, and their intentions in case of different unit profits were calculated. The values in Table 2 represent the percentage of maximum waste disposal capacity which 3Ps are willing to provide under different unit profits. The values are multiples of 5% according to the questionnaires.

Only eight 3Ps have the relevant qualifications to operate in Guangzhou. Owing to the clear statistical data, the look-up table function in Vensim was directly used to represent this rate variable. The numerical relationship between the unit profit of the 3P and its intention to engage in waste disposal was obtained by the following equation, and the results are shown in Table 3.

$$Intention\;of\;the\;3P\;to\;engage\;in\;waste\;disposal\;=\overset{8}{\underset{n=1}{\sum }}Max\;disposal\;capacity\;of\;n\times the\;value\;in\;the\;corresponding\;unit\;profit\;of\;n\;$$

where, *n* stands for the number of the 3Ps in Table 2.

The descriptions of other variables in this module are shown in Table 4.

**Figure 2:**
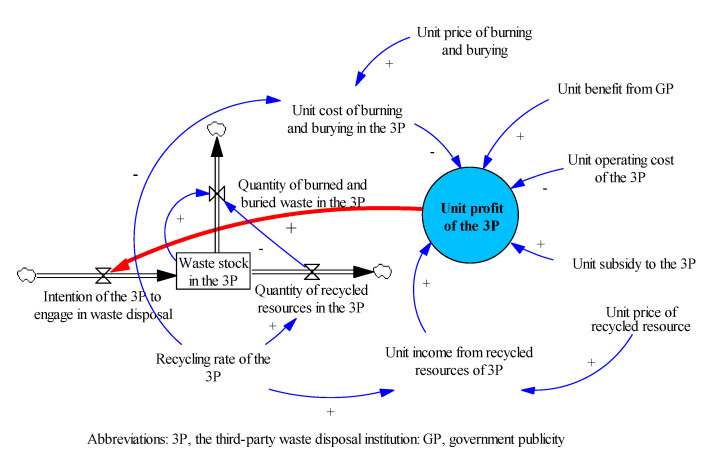
The CLD of 3P Waste disposal module.

Once urban waste has been generated, part of it is outsourced to the 3Ps. Part of the waste is converted into recycled resources, and the rest is burned and buried. The costs of the 3Ps include operating costs, and burning and burying costs, and their incomes mainly come from government subsidies and the recycled resources. Government publicity (GP) enables residents to better classify waste, which reduces the cost of waste disposal. The CLD of the 3P waste disposal module is shown in Figure 2.

The effects of the feedback loops are described now. According to the profit-seeking nature of the enterprise, the higher the unit profit of the 3P is, the higher is its intention to attend to waste disposal [60]. The intention of the 3P to engage in waste disposal turns into waste stock. And as the waste stock increases, the quantities of waste burned and buried as well as recycled resources increase. The former increases the unit cost of burning and burying, which reduces the unit profit of the 3P. The recycled resources increase the income for the 3P, thus increasing its unit profit. 

#### 3.3.2. SO Waste Disposal Module

The SO is a public welfare institution operated by the government. Although it needs market-oriented reform, it cannot be responsible for its own profits and losses, and there is a large gap between its income and cost that needs to be compensated for by the government in the form of subsidies.

The core variable in this module is the gap between the income and cost in the SO, which determines the amount of government subsidies afforded to it. The equation can be described as follows:

(1) Gap between income and cost in the SO = Income from recycled resources of the SO + (Unit benefit from GP—Unit operating cost of the SO) × Quantity of waste disposed by CD—Cost of burning and burying in the SO

In the above equation:

(2) Cost of burning and burying in the SO = Unit price of burning and burying × (Quantity of waste burned and buried by CD + Quantity of waste burned and buried by AD)
(3) Income from recycled resource of the SO = Unit price of recycled resource × (Quantity of resources recycled by CD + Quantity of resources recycled by AD)

**Figure 3:**
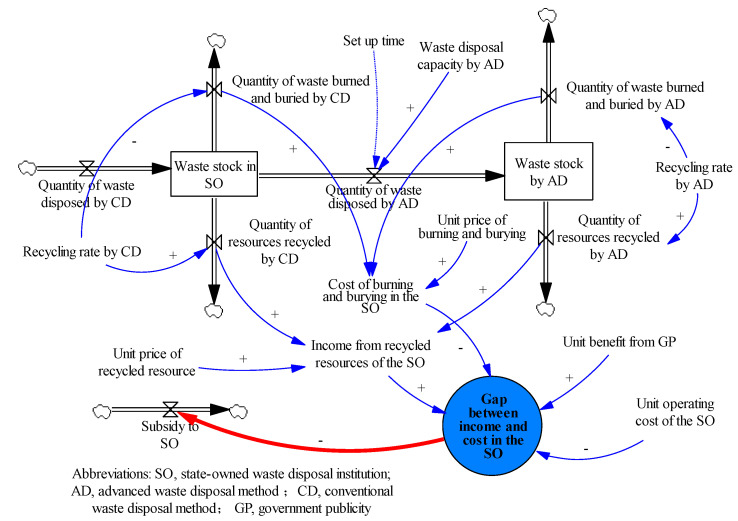
The CLD of SO waste disposal module.

The other variables in this module are similar to the 3P waste disposal module. The CLD of the SO waste disposal module is shown in Figure 3. Owing to the limited waste disposal capacity of the 3Ps, the SO has to undertake the remaining waste disposal work as the protector of the urban environment. The process of disposal of waste in the SO is similar to that of the 3P. The increasingly serious dilemma of “waste siege” shows that the conventional method of waste disposal (CD) of the SO needs to be improved. Through investment in R&D, SOs can purchase advanced machinery, improve disposal technology, obtain the disposal capacity by the advanced method of waste disposal (AD), and convert more LVRWs into recycled resources to reduce the proportion of burning and burying. However, an upgrade to advanced capacity of waste disposal cannot happen overnight. It requires a circular process of “investment–income–reinvestment.”

#### 3.3.3. Government Subsidy Policy Module

This module describes the impact of the government’s four subsidy policies on the UWRS. 

(1) Policy #1

Policy #1 involves outsourcing subsidies to the 3Ps. The government gives the 3P a certain amount of subsidy for each ton of waste disposed. Policy #1 occupies reasonable proportion of the SCP. The amount of subsidy should solicit sufficient intention of the 3P to engage in waste disposal, but should not leave the disposal capacity by AD in the SO idle. 

The description of the equation for Policy #1 is:

*Policy #1 = Unit subsidy to the 3P × Intention of the 3P to engage in waste disposal*

The expression of intention of the 3P to engage in waste disposal refers to the 3P waste disposal module.

(2) Policy #2

Policy #2 involves subsidy to the SO, and is a necessary subsidy. Before LVRWs are fully reused, the SO cannot fill the gap between its income and cost, and the government needs to subsidize it to guarantee its normal operation. The description of the equation for Policy #2 is:

*Policy #2 = IF THEN ELSE (-Gap between income and cost in the SO ≥ SCP except subsidy for Policy #1, SCP except subsidy for Policy #1, MAX(0,—Gap between income and cost in the SO))*

This is a conditional function that defines the amount of subsidies under different conditions:

**Condition** **1**. *After the government has subsidized the 3Ps, the remaining SCP is insufficient to fill the gap between the income and cost in the SO. Then, all the SCP is subsidized without credit. This has a serious impact on the normal operation of the SO, and the government should try to avoid this situation when designing the subsidy mechanism.*

**Condition** **2**. *After the government subsidizes 3Ps, the remaining SCP is sufficient to fill the gap between the income and cost in the SO. Then, the amount of subsidy is the value of this gap.*

**Condition** **3:** *When the value of the gap between income and cost is negative, that is, when the SO begins to make a profit, the subsidy is zero. From then on, the SO is responsible for its own profit and loss.*

(3) Policy #3

Policy #3 is a subsidy for the R&D of GT for waste disposal. The subsidy can help continually improve the UWRS. It can improve the comprehensive recycling rate of the SO and force 3Ps to upgrade their waste disposal technologies. After completing Policy #1 and Policy #2, the remaining subsidies are allocated to Policy #3 and Policy #4 according to the proportion set by the government. Note that all policies are rate variables; the government provides subsidies of Policy #3 every week, but Policy #3 cannot be applied directly to the UWRS. What is applied to UWRS is the total R&D investment for GT, which is the stock variable of Policy #3. There is a process from quantitative changes to qualitative changes in R&D investment. The role of Policy #3 in the UWRS has two facets. First, Policy #3 is directly related to waste disposal capacity by AD in the SO and conforms to the law of diminishing marginal returns [39,40]. According to the statistics of Guangzhou’s R&D investment on GT for waste disposal from 2010 to 2020, the relationship between Policy #3 and waste disposal capacity by AD in the SO can be obtained as follows:

(1) $$\mathrm{Waste}\mathrm{disposal}\mathrm{capacity}\mathrm{by}\mathrm{AD}=\ln\left(\frac{Total\;R\&D\;investment\;for\;GT}{1\times 10^{7}}+1\right)\times 10,000$$

Because a certain period to prepare the investment is required between R&D and the relevant implementation:

*Quantity of waste disposal by AD = DELAY1(Waste disposal capacity by AD, Set-up time)* (2)

The other role of Policy #3 in the UWRS is to reduce operating cost by improving the automation and intelligence of the SO. R&D investment to reduce operating cost conforms to the law of the learning curve [61,62,63]. In this paper, Wright’s formula [64] is used to describe the learning effect, that is, the change in operating cost with total R&D investment in GT:

(3) $$C_{t}=C_{0}\times {\left(\frac{I_{sum}}{I_{0}}\right)}^{-a}$$

(4) $$a=-\frac{\ln R}{\ln2}$$

$C_{t}$ is the operating cost of the SO at time t, $C_{0}$ is the initial operating cost, $I_{sum}$ is the total R&D investment on GT at time t, $I_{0}$ is the initial investment, a is the learning coefficient, and R is the rate of decline in cost. 

When the rate of decline in cost is mainly affected by the proficiency of the machines, the value of R is higher, and was set to 90% according to previous research results. $C_{0}$ is 95 CNY/ton according to the current average unit operating cost of the SO in Guangzhou, and $I_{0}$ is 1 million CNY according to initial value of Policy #3. The equation of cost under the action of Policy #3 can thus be obtained:


(5) $$\mathrm{Unit}\mathrm{operating}\mathrm{cost}\mathrm{of}\mathrm{the}\mathrm{SO}=95\times {\left(\frac{Total\;R\&D\;investment\;for\;GT}{1,000,000}\right)}^{\frac{\ln0.9}{\ln2}}$$

(4) Policy #4

Policy #4 is the subsidy for government publicity (GP), such as waste classification and green consciousness. GP can help reduce the cost of waste disposal. The effects of the methods of publicity are ranked by order of importance as economic subsidy > mobile phones and networks > laws, and regulations > community publicity [65,66]. In general, more effective publicity methods yields higher costs. Based on survey data for Guangzhou, the positive impact of residents’ response to waste disposal under different GP subsidies was surveyed. After quantitative processing, Table 5 was obtained.

Table 5 shows that the relationship between the unit benefit from GP and Policy #4 generally conforms to the law of diminishing marginal return. To express the relationship between them, this paper uses a logarithmic function for fitting:

$$Y=C_{1}\times \ln\left(\frac{X}{C_{2}}+1\right)$$

where Y represents unit benefit from GP, X represents the subsidy for GP (Policy#4), and $C_{1}$ and $C_{2}$ are the fitting coefficients. The results of fitting are shown in Table 6.

The results show that: (1) fitness R-squared = 0.99 > 0.8, and (2) Durbin–Watson stat = 2.15, in the interval (1.5, 2.5). This proves that the residuals had no sequential correlation. (3) The value of Prob. approached zero. The fitting coefficients passed the test of significance. (4) The SE of regression/mean dependent var. = 1.85/64.29 = 2.88% < 15%. The test of standard deviation was successfully passed.

The results of fitting all passed the above testes, and the equation relating the variables is as follows:

$$\mathrm{Unit}\mathrm{benefit}\mathrm{from}\mathrm{GP}=78.3\times \ln\left(\frac{Policy\;\#4}{3185291}+1\right)$$

**Figure 4:**
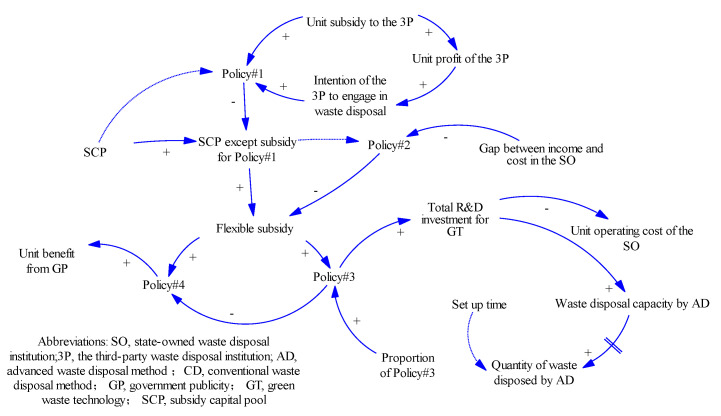
The CLD of the government subsidy policy module.

The relationship between subsidies and their objectives is described in Figure 4. First, the subsidy to 3P has the highest priority. Second, the income from recycled resources cannot cover the cost of the SO, thus, the formal operation of the SO relies heavily on subsidies. Third, if there is a surplus in the SCP, other subsidies are flexibly distributed to GT and GP. According to the analysis, the priority of the subjects of subsidy in this module is 3P > SO > GT = GP. 

### 3.4. SD Model of the UWRS

This paper used data on urban waste disposal in Guangzhou, from the Guangzhou Municipal Bureau of Statistics, from 2019 to 2020 as source data for the simulations. Some of the data were obtained after processing the original data. The sources are shown in Table 7.

**Figure 5:**
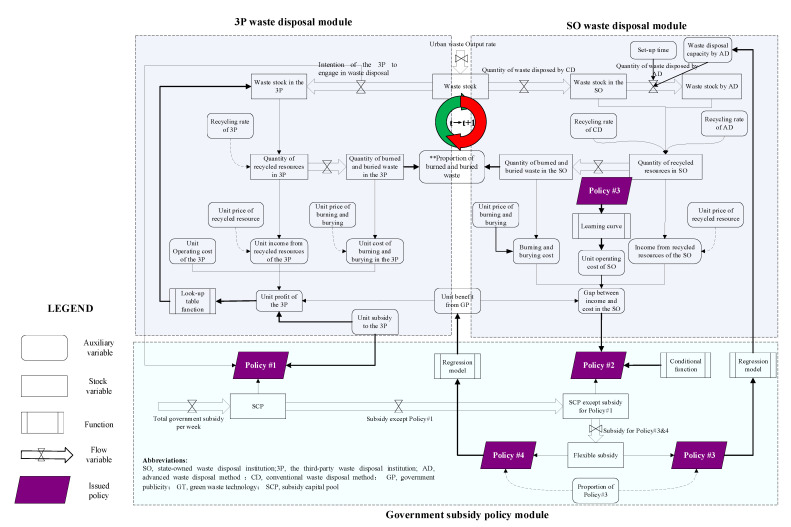
SD model of UWRS.

The SD model of the UWRS can be established by integrating the above three modules. This is shown in Figure 5. 

## 4. Results and Analyses

The codes for the main variables used here are shown in Table 8.

### 4.1. Model Implementation and Validation

The model was implemented on Vensim PLE version 8.2.1 (https://vensim.com/vensim-personal-learning-edition/ accessed on 15 September 2021). The basic parameters of the simulation were as follows: initial time = 0, final time = 50, time step = 1, and unit of time = week.

The values of the variables in the model were taken from the official statistics of Guangzhou, and the process of waste disposal in the model was identical to the actual operation. Policy #1 and Policy #2 encapsulate the laws and regulations issued by Guangzhou, respectively, in 2009 and 2017. Policy #3 and Policy #4 in the model are policies that need to be validated. Therefore, validation involved observing the outputs of the system when Policy #3 and Policy #4 were not applied. The flexible subsidy in the model was kept at zero, and the *P_bb_* in the system was 86.3% as obtained in the simulation. The actual value was 87.19% for Guangzhou in 2020. Thus, the difference was within a reasonable range, and the SD model passed the validation test.

### 4.2. Simulation Results

Several scenarios were run to verify the roles of different policies, as follows:

**Scenario** **1**. *All four policies worked simultaneously. All the variables were set according to*Table 5.

**Scenario** **2**. *Policy #1 was not used. At this time, the unit subsidy to the 3P was set to zero, and the other settings were the same as in scenario 1.*

**Scenario** **3**. *Policy #3 was not applied. The proportion of Policy#3 was set to 0, and the other settings were the same as in scenario 1.*

**Scenario** **4**. *Policy #4 was not applied. The proportion of Policy #3 was set to 1, and the other settings were the same as in Scenario 1.*

Policy #2 was a necessary subsidy and worked in any scenario.

**Figure 6:**
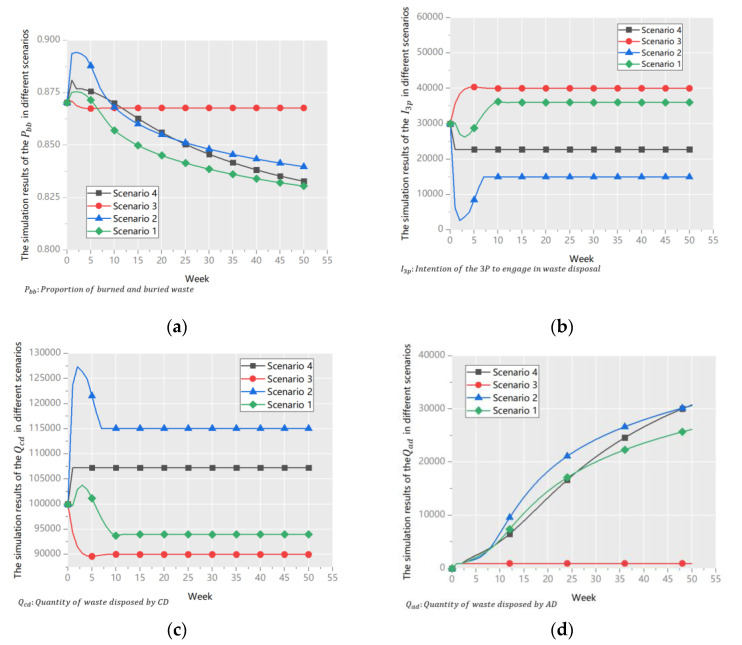
The simulation results of the important indicators in different scenarios:(**a**) *P_bb_*; (**b**) *I_3p_*; (**c**) *Q_cd_*; (**d**) *Q_ad_*.

Under the four scenarios, the important indicators in the UWRS model, *P_bb_*, *I_3p_*, *Q_cd_*, and *Q_ad_*, were compared. The results are shown in Figure 6. Note that:

*P_bb_ = (Quantity of burned and buried waste in the SO + Quantity of burned and buried waste in the 3P)/Urban waste output rate.*

In Scenario 1, *P_bb_* was the lowest, that is, the performance of the UWRS was the best (Figure 6a). This shows that the multiple policies complemented one another, this finding is consistent with previous studies [25,41,46]. Therefore, the way of improving the UWRS is to optimize the proportion of policies and formulate the scheme of coordination of each.

In Scenario 2, *I_3p_* was the lowest (Figure 6b). Without the subsidy, the profits of the 3P could not meet the requirements of complete marketization [11]. Owing to a fixed SCP, canceling Policy #1 increased the flexible subsidy, which meant a greater subsidy to GT; thus, *Q_ad_* was the highest in all scenarios (Figure 6d). However, a lower *I_3p_* led to more pressure on the SO (Figure 6c), that finally led to unsatisfactory performance of the UWRS (Figure 6a).

In Scenario 3, without investment in GT, *Q_ad_* remained constant (Figure 6d). The competitiveness of the SO was low (Figure 6c), and urban waste disposal relied heavily on the 3Ps (Figure 6b). According to Hypothesis 3, the 3Ps did not take the initiative to upgrade their technologies when there was little competitive pressure; therefore, in the long run, Guangzhou’s UWRS could not be continually improved [27]. Figure 6a shows that the performance of the UWRS was the worst in all scenarios. The time-down function shows that after 50 weeks, the difference between values of *P_bb_* in scenarios 3 and 1 was as high as 4.9%.

In Scenario 4, without investment in GP, residents did not pay attention to waste classification, which increased the cost of waste disposal. This finding is consistent with previous studies [24,45]. Due to the cost sensitivity of the 3P, *I_3p_* significantly decreased. Figure 6b shows that the value of *I_3p_* in scenario 4 was higher only than that in Scenario 2, which involved no subsidy to the 3Ps (Figure 6b). However, because Policy #3 and Policy #4 shared a flexible subsidy, the subsidy to Policy #3 increased when Policy #4 was canceled, and the system performed better in the long term (Figure 6a). Appropriately using Policy #4 needs to be further studied.

### 4.3. Sensitivity Analysis

We have shown that the UWRS is the most efficient under the combined action of the four policies. Therefore, based on scenario 1 above, we examine whether the efficiency of the system continues to improve when some important auxiliary variables are changed:

(1) Total government subsidy per week (*S_tg_*)

**Figure 7:**
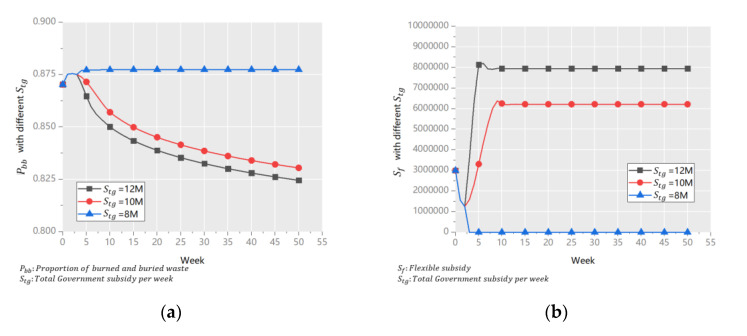
*P_bb_* (**a**) and *S_f_* (**b**) with different *S_tg_*.

The government expects subsidies to play the biggest role in the UWRDS. On the premise of a fixed SCP, it needs to know the minimum *S_tg_* to ensure the stability of the UWRS, and the rate of improvement in the system’s performance with the increase in subsidies. Therefore, we set *S_tg_* to 8 million (M), 10 M, and 12 M, for sensitivity analysis. The results are shown in Figure 7.

Figure 7a shows that when *S_tg_* = 8 M, the system’s efficiency was the lowest, and there was no trend of improvement. When *S_tg_* = 10 M and *S_tg_* = 12 M, the system’s efficiency improved at the same rate. The reason is illustrated in Figure 7b. When *S_tg_* = 8 M, *S_f_* = 0. This shows that 8 M could not meet the requirements of the 3Ps and the SO. In addition, the values of *P_bb_* at the end of the simulation period were 82.48% and 81.74% when *S_tg_* was 10 M and 12 M, respectively. The gap was small such that the government needed to consider whether the extra 2 M/week was worth the benefit.

The conclusion drawn from the results are quite different from previous studies. Most previous studies showed that as long as the *S_tg_* were distributed reasonably to specific paths, *S_tg_* could play an effective role in waste disposal [15]. While the results in this paper prove that in the UWRS, the marginal benefit decreases immediately after *S_tg_* reaches the threshold. This is because the recovery rate of LVRW is affected by many factors and lags behind the *S_tg_* input. Excessive S_tg_ in a short time can not significantly improve the recovery rate, and the return on *S_tg_* is very low.

(2) Unit subsidy to the 3P (*S_3p_*)

**Figure 8:**
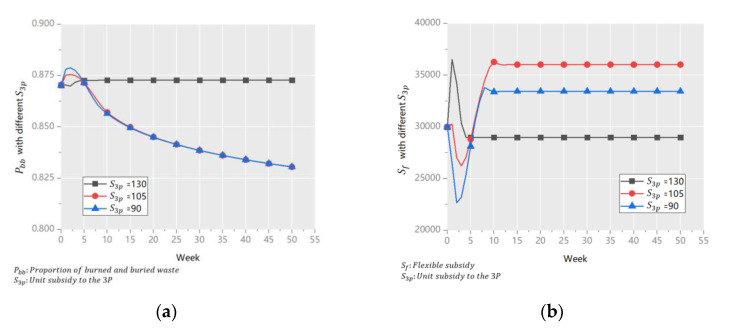
*P_bb_* (**a**) and *S_f_* (**b**) with different *S_3p_*.

*S_3p_* is a very important variable that is directly related to the unit profit of the 3P and *I_3p_*. We adjusted its value based on an initial value to 90, 105, and 130, and the results are shown in Figure 8.

Figure 8a shows that at the end of the simulation period, *P_bb_* was the same when *S_3p_* = 90 and *S_3p_* = 105, but was the highest when S_3p_ = 130. Highest *S_3p_* means that the system’s performance was the worst. The reason is shown in Figure 8b. When *S_3p_* = 130, *I_3p_* increased in the first two weeks owing to the high subsidy, and rapidly decreased in the next three weeks because too many subsidies were given to the 3Ps in place of the subsidy for GP, and the cost of waste disposal increased. The results prove that *S_3p_* and system performance are not positively correlated, and the set values of *S_3p_* were excessively high.

The results shows that *S_3p_* is an important indicator for the incentive *I_3p_*, this finding is consistent with that of Liu (2021) [15] and Shi (2020) [67]. Furthermore, it is proved that an excessively high *S_3p_* hindered waste classification and reduced the unit profit of the 3Ps in this paper. An excessively high *S_3p_* hindered waste classification and reduced the unit profit of the 3Ps. In addition, *S_3p_* in Guangzhou is a slightly higher, and can be reduced appropriately such that it does not affect the efficiency of waste recycling over the long term. However, owing to the currently low *I_3p_* in China, the current subsidy can be maintained, and once enough 3Ps have been attracted, *S_3p_* can be reduced gradually.

(3) Proportion of Policy #3 (*P_#3_*)

**Figure 9:**
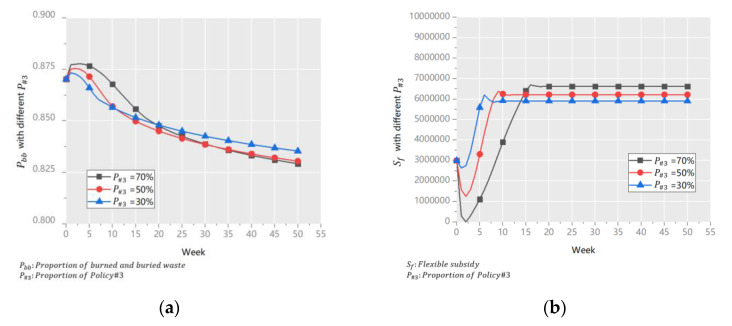
*P_bb_* (**a**) and *S_f_* (**b**) with different *P_#3_*.

Policy #3 and Policy #4 share the *S_f_*, and *P_#3_* is the proportion of the flexible subsidy to Policy #3. *P_#3_* was set to 30%, 50%, and 70%, and the results are shown in Figure 9.

Figure 9a shows that in the first eight weeks, the system’s performance rankings were 30% > 50% > 70%. But at the end of the simulation, the order was the opposite. This shows that the short-term impact of GP was more significant while the long-term impact of GT was. Figure 9b shows that the value of 70% ensured the *S_f_* to maintain in a high value. A bigger *S_f_* and a higher *P_#3_* implied more R&D investment for GT, which is the driving force for sustainable improvements in the UWRS.

Daniel [68] and Wang [69] respectively proved the positive role of GT and GP in waste disposal, and Diggle et al. [25] proved that the performance of the mixed policy is better than that of both single policies. This article further holds that the response period should be considered when mixed subsidy policies are implemented. A policy with a short response period usually has weak long-term effects, and vice versa. Therefore, on the premise that the government’s SCP is limited, decision makers should not only solve the problem of urban waste, but also reasonably plan for the sustainable development of the urban environment. For example, in this paper, although the return on GT investment was high, investment in it cannot be prioritized over operational subsidies for the SO and 3P because it has a long response period. Instead, decision makers should design a long-term circular development mechanism of “input–implementation–output–reinput.”

## 5. Discussion

### 5.1. Policy Implications

The results here have shown that the effects of mixed subsidy policies on the UWRS are unequal to the sum of those of the individual policies. The principle of policy design is not only to encourage individual subjects play their unique roles in the UWRS, but also to avoid a conflict of interest between them. This result is consistent with that of Dhanshya et al. [25], who compared the effects of both single policies and mixed policies on plastic waste mitigation in India. Zhou et al. [15] reached a similar conclusion when studying how government subsidies affect the strategic choices of enterprises and individuals in agricultural waste recycling. Furthermore, our study explicitly demonstrates the causal paths leading to this difference in effects when multiple policies are simultaneously implemented. Based on the results above, the following policy implications are proposed:

First, the design of a subsidy policy should be targeted at bringing the market mechanism into the UWRS. If waste disposal is regarded only for environmental protection and as a public welfare, waste disposal cannot be continually improved. Subsidy policies can passively fill the gap between the operating costs and incomes of such tasks but should be used to push recyclables in urban waste forward to the market. In previous studies, it has been considered a reasonable path to build a closed-loop supply chain of the recyclables through market mechanism [70,71]. Based on previous studies and the status quo of China’s city, this paper proposes a competitive subsidy strategy and designs reasonable paths to implement it: (1) encouraging competition among 3Ps by setting a fixed subsidy and waste disposal capacity, (2) encouraging competition between 3Ps and the SO by introducing a subsidy distribution standard, and (3) providing incentives for enterprises using recyclable resources [72].

Second, the government should enable users of advanced GT to play a biggest role in the UWRS. GT has the greatest potential to improve system performance. Previous studies have proved the importance of GT for waste recycling [69,73], but most of the policies designed to promote GT development are static. They are not considered that the development of GT requires the accumulation of funds, and its influence is also dynamic and gradually released. Because R&D for GT requires a large investment, the response period of the subsidy for GT is long. In the initial stage, with a low GT level, the government could enhance both the filtering criteria and subsidies to attract 3Ps that own the advanced GT and have a high rate of waste recycling. With the accumulation of R&D investment in GT, waste disposal capacity by the AD in the SO gradually increases. At this stage, the subsidy to 3Ps should be appropriately reduced to discourage its engagement in waste disposals, so that advanced waste disposal capacity in the SO is not rendered idle. Finally, when the effect of GT decreases owing to diminishing marginal benefit, the subsidy for GT should be suspended until a revolutionary new GT emerges; at this stage, the diffusion of the existing GT should be the focus of the government’s task.

Third, the government should make “a ratchet effect” among subjects in the UWRS. Most previous studies focused on the incentive role of subsidies [11,38,41], but ignored their guiding role. Combined with the sunk cost theory, this paper holds that the subsidy policy should attract multiple subjects to participate in waste recycling when the LVRW is not fully market oriented. The government should not make each subject too reliant on subsidies. If all subjects are expected to give full play to their creativity, it is necessary to invest a lot of sunk costs in the process of waste disposal. Based on this theory, the government should encourage 3Ps to carry out technological upgrades through joint R&D with it, and continually expand advanced waste disposal capacity using enterprise income. In addition, government should support special labs in scientific research institutions to maintain the R&D of GT.

### 5.2. Model Adaptation

Although the SD model was developed based on the urban situation in China, the framework of the model can be applied other countries to improve their systems of waste disposal through appropriate adaptations. There are four considerations for model adaptation:

(1) The priority of the subjects of subsidy in the subsidy policy modules is as follows: 3P > SO > GT = GP. The order of priorities can be adjusted according to the empirical situation. If there is another subject in the research, it can be added to the flexible subsidies, and its initial proportional distribution can be confirmed by the dilemma that the local government is confronted with.

(2) In this paper, a regression model was used to express the impact of Policy #4 on profits from GP and Policy #3 on waste disposal capacity in the AD. If more accurate data are available in a given context, the look-up table function in Vensim can be used to improve the model. We refer here to the expression for the relationship between Policy #1 and intention of the 3P to engage in waste disposal.

(3) This paper used the credit management of 3Ps. In practice, 3Ps may be able to falsely report their waste disposal capacities and defraud the government of subsidies due to problems with measuring this capacity in different countries or cities. Therefore, according to the given situation, two variables can be added to the 3P waste disposal module, the probability of fraud by 3Ps and the consequences in case cheating is exposed, to ensure compliance with law.

(4) At present, the subsidy to the SO fills the gap between cost and income in the SO. This creates the risk of the SO becoming lazy and not innovating. To inspire innovation in the SO, the government can choose to subsidize only a certain proportion of the cost–benefit gap, and the residual gap can be dynamically subsidized according to waste disposal performance. In the SO waste disposal module, the condition function can be set to implement the above modification.

## 6. Conclusions and Future Work

To solve the problem of urban waste caused by a high proportion of LVRWs and a poor recovery rate, this paper established an SD model of mutual supplement between the SO and 3Ps and designed an incentive mechanism composed of four subsidy policies (subsidy to the SO, subsidy to the 3Ps, subsidy for GT, and subsidy for GP) under the condition of a fixed total SCP. By simulations based on the relevant data for Guangzhou, problems in current subsidy policies in the city were identified. An effect analysis of mixed subsidy policies was used to determine the optimal set of subsidy schemes. Some notable results can be summarized as follows:

(1) We proved that there is a joint force between multiple subsidy policies. If any of them is removed, the performance of the UWRS worsens. We also proved that diversified subject participation is key to the sustainable development of the UWRS.

(2) There are two problems in current subsidy policies in Guangzhou: The total subsidy is slightly high, which leads to a waste of government finance. And the unit subsidy to the 3P is too high, which makes them reliant on it and unable to operate independently. Therefore, on the premise of appropriately reducing the total subsidy, the proportion of participation of the SO and 3Ps in the UWRS can be adjusted by redesigning the subsidy policy. A specific recycling rate as the subsidy threshold should be set so that the government can implement the scheme of “no subsidy for unqualified 3Ps and more subsidy for excellent 3Ps,” and encourage 3Ps to attend to the recycling rate of LVRWs. In addition, with improvements in waste disposal capacity due to the use of advanced methods in the SO, the subsidy threshold of 3Ps should also dynamically rise.

(3) The effect of GP has short-term advantages and significant long-term potential. The government can allocate subsidies more flexibly according to the different effects of policies. For example, the current urban environment has reached a critical situation due to the problem of waste, and the subsidy policy should be inclined to GP to strive for significant improvements in the short term. On the contrary, when the current urban waste disposal situation is satisfactory temporarily, decision makers can pay attention to the R&D of GT to maintain long-term sustainable development.

There are some limitations related to the policies in the UWRS model that may introduce some degree of uncertainty to our analysis. First, in Hypothesis 3, it was assumed that the recycling rate of the 3P was fixed, which means all 3Ps were assumed to be at the same technical level. This hypothesis is reasonable in short time in China. In long time, with the development of coordination mechanism among the subjects in UWRS, 3Ps could make more profits from the waste disposal and wish to improve recovery rate further. There are also some other countries where LVRW waste management is based primarily/only on the 3Ps. In either case, 3Ps will face the choice of whether to take the lead in investing in GT or adopting the “free rider” behavioral mode. The decision depends on government policies, market environment, and operational efficiency. In future work, we will use evolutionary game theory to study the conditions for the balance in investment in R&D among 3Ps and determine the optimal path for 3Ps at different scales to improve the recycling rate under the given government subsidy policy. Second, with the integration of the market mechanism, more subjects will participate in the UWRS, for example, financial institutions and renewable resource utilization enterprises. In the future, we will add new modules to the UWRS model, study the cross-action mechanism of multiple subjects, and design new policies to ensure the collaboration among them.

## Figures and Tables

**Table 1:** Comparing current study with previous studies in implementation of incentive mechanism.

Country	Incentive Subject	Incentive Mechanism	Description/Result	Reference
Incentive Mechanism for Recycling Recyclable Waste				
U.K	Recycling Companies	Recycling Subsidy	It used a two-level programming model to analyze the government’s optimal strategy for subsidizing recycling.	[38]
China	Recycling Companies	Profit Compensation Mechanism	In order to achieve a balance between corporate profits and social responsibilities, the government coordinates the reverse supply chain through a profit compensation mechanism.	[39]
Europe	Manufacturing Companies	Reward & Punishment	The government’s reward and punishment can guide producers to give full play to their extended responsibility. Inspired by the subsidy, producers will vigorously carry out the construction of reverse logistics supply chains, innovate technology for green product designs, and improve their resource reuse efficiency.	[40]
China	Manufacturing Companies	Subsidies	It analyzed the impact of four kinds of subsides on circular development: initial subsidy, recycling subsidy, R&D subsidy, production subsidy.	[41]
Bangladesh	Customers &Recycling Agencies	Tax Credits	Tax credits subsidy is given to agencies who are assigned to collect e-waste and consumers to encourage them in order to bring their e-waste for recycling. However, it imposes an additional burden on both from the financial and administrative side of the Government	[42]
Iran	Manufacturers & Retailers	Tax exemption and subsidy	It compared the government’s role in providing different incentive strategies (tax exemption and subsidy) for closed-loop supply chain members	[43]
USA	Customers, Manufacturers & E-NGOs	Tax	It compared the government mechanisms of imposing taxes on manufacturers and setting recovery rates as penalty targets. The results showed that the choice of mechanism varies according to the perspective of the stakeholder.	[44]
Incentive Mechanism for Recycling LVRW				
China	Resident	Reward	Appropriate subsidies can guide residents to recycle LVRW, which can alleviate the problem of low rate of LVRW.	[45]
Czech Republic	Resident	Reward	It explored that implementing a reward subsidy program can double the existing paper and plastics separation rates.	[24]
India	Resident	Mixed policies	It simulated four major policy interventions and explored that while composite combinations of policies offer more effective policy mix than individual policy interventions, a suitable choice of policy mix along with its timing and extent is crucial.	[25]
Canada	Manufacturing Companies	Pricing Schemes	In order to reduce single-use plastic (SUP) waste, the EPR system is implemented and pricing subsidies are adopted to increase the waste recovery rate.	[26]
China	Recycling Companies	Special Subsidy	It explored the special subsidy which can be carried out directly (assistance payment) or indirectly (subsidize R&D projects, etc.).	[11]
China	3Ps	Processing Subsidies	According to the amount of LVRW recycled and reused, 3Ps obtain processing subsidy by the government, which effectively promoting the recycling and classification of LVRW by 3Ps.	[46]
China	Recycling all Participants	Mixed Subsides	Aiming at the best recovery efficiency, this paper discusses a mixed subsidy policy model on the basis of considering the government fixed subsidy capital pool.	Current study

**Table 2:** The intention percentage of max disposal capacity of the 3P in different unit profits.

*No.* *Unit Profit*	*1*	*2*	*3*	*4*	*5*	*6*	*7*	*8*
100	0.2	0	0.05	0	0.05	0	0.1	0.05
200	0.3	0.2	0.1	0.3	0.1	0.2	0.2	0.2
300	0.4	0.3	0.2	0.5	0.3	0.35	0.4	0.4
400	0.55	0.5	0.4	0.75	0.4	0.55	0.55	0.7
500	0.75	0.7	0.65	1	0.6	0.7	0.75	1
600	1	1	1	1	1	1	1	1
Max capacity	30	10	60	15	20	25	20	20

**Table 3:** Intention of the 3P to engage in waste disposal in different unit profits.

**Unit profit of 3P (Chinese Yuan, CNY)**	0	100	200	300	400	500	600
**Intention of the 3P to engage in waste disposal (thousand tons/week)**	0	13	36.5	65.25	103.5	148	200

**Table 4:** Equation description of variables.

Variable	Equation Description
Unit profit of the 3P	Unit subsidy for the 3P + Unit income from recycled resource in the 3P + Unit benefit from GP − Unit operating cost of 3P − Unit cost of burning and burying in the 3P
Unit income from recycled resource in the 3P	Unit price of recycled resource × Recycling rate of 3P
Unit cost of burning and burying in the 3P	Unit price of burning and burying × (1 − Recycling rate of 3P)
Unit benefit from GP	Refer to the government subsidy policy module
Waste stock in the 3P	∫ (Intention of the 3P to engage in waste disposal—Quantity of waste burned and buried in the 3P -Quantity of recycled resource in the 3P)
Quantity of recycled resources in the 3P	Waste stock in the 3P × Recycling rate of the 3P
Quantity of burned and buried waste in the 3P	Waste stock in the 3P − Quantity of recycled resources in the 3P

**Table 5:** Statistics of the unit benefit from GP.

***Policy#4 (CNY)***	***0***	***1M***	***2M***	***3M***	***4M***	***5M***	***7M***	***10M***
**Unit benefit from GP (CNY)**	0	20	37	52	64	74	94	109

**Table 6:** The fitting results.

Coefficient	Results	Std. Error	*t*-Statistic	Prob.
$C_{1}$	78.25234	6.006924	13.02702	0.0000
$C_{2}$	3185,291	395,232.7	8.059280	0.0005
**Main fitting test parameters**	**Results**			
R-squared	0.997052			
Adjusted R-squared	0.996462			
S.E. of regression	1.854672			
Mean dependent var	64.28571			
Durbin-Watson stat	2.153398			

**Table 7:** Data sources.

*Variable*	*Values*	*Unit*	*Data Sources*
(1) Urban waste output rate	0.13 million	Ton/week	Mean value of weekly waste output data in Guangzhou from 2019 to 2020.
(2) Unit price of burning and burying	56	CNY/ton	According to the urban waste disposal report of Guangzhou in 2020, the costs of burning and burying are 145 CNY/ton and 21 CNY/ton respectively, with a proportion of 2:5. The value in this paper is the weighted average value.
(3) Unit operating cost of the SO	80	CNY/ton	Guangzhou statistical yearbook
(4) Unit operating cost of the 3P	60	CNY/ton	Average value of 8 3Ps
(5) Unit price of recycled resource	600	CNY/ton	The main components of LVRW are wood chips, cartons and other recycled resources, and the weighted average value is taken according to the proportion and the price of different recycled resource.
(6) Set up time	4	week	Practical experience
(7) Unit subsidy for the 3P	105	CNY/ton	Guangzhou statistical yearbook
(8) Government total subsidy per week	10 million	CNY	Guangzhou statistical yearbook
(9) Recycling rate by CD	10%	Dmnl	Guangzhou statistical yearbook
(10) Recycling rate of the 3P	20%	Dmnl	Average value of 8 3Ps
(11) Recycling rate by AD	30%	Dmnl	According to the data of Guangzhou statistical yearbook in 2020, 14% of the total waste in Guangzhou is directly recyclable, and 38% is the LVRW [8]. According to international advanced standards, the highest recovery rate of direct recyclable part of waste is 80%, and the highest recovery rate of LVRW is 50%. It can be calculated that Recycling rate by AD = Direct recyclable × its highest recovery rate + LVRW × its highest recovery rate = 14% × 80% + 38% × 50%≈30%
(12) Proportion of Policy#3	50%	Dmnl	Initial value

**Table 8:** Code of main variables.

Code	Variable
*I_3p_*	Intention of the 3P to engage in waste disposal
*P_#3_*	Proportion of Policy#3
*P_bb_*	Proportion of burned and buried waste
*Q_cd_*	Quantity of waste disposed by CD
*Q_ad_*	Quantity of waste disposed by AD
*S_tg_*	Total Government subsidy per week
*S_f_*	Flexible subsidy
*S_3p_*	Unit subsidy to the 3P
